# Metareasoning: Theoretical and Methodological Developments

**DOI:** 10.3390/jintelligence13010005

**Published:** 2025-01-03

**Authors:** Linden J. Ball, Beth H. Richardson

**Affiliations:** School of Engineering & Computing, University of Central Lancashire, Preston PR1 2HE, UK; bhrichardson@uclan.ac.uk

## 1. Introduction

This Special Issue aims to capture current theoretical and methodological developments in the field of metareasoning, which is concerned with the metacognitive processes that monitor and control our ongoing thinking and reasoning. Monitoring processes evaluate the efficacy of the “object-level” cognition that underpins task performance (Nelson and Narens 1990), including processes that are involved in understanding task information, drawing inferences and making decisions. In contrast, control processes allocate cognitive resources (e.g., attention and working memory), initiate new strategies and also terminate failing ones.

Progress in understanding metareasoning was given significant impetus several years ago, when Ackerman and Thompson (2017) published an important article in which they reviewed the state of the art in research and theorising relating to the metacognitive monitoring and control that arises in situations involving high-level cognition. A key outcome of this review was Ackerman and Thompson’s presentation of a “metareasoning framework” used to integrate existing findings relating to metacognition in situations involving thinking and reasoning. Central to this framework is the idea that metacognitive monitoring takes places continually during task performance and is sensitive to fluctuating feelings of certainty and uncertainty that are experienced by a reasoner regarding how well an object-level process or strategy is unfolding. Likewise, metacognitive control continually responds to these shifting levels of experienced certainty or uncertainty—maintaining ongoing object-level processing if it is going well or else triggering strategy change if it is floundering. Metacognitive control processes are also needed to cease object-level processing if a satisfactory outcome is achieved or if object-level processing is failing to deliver an outcome.

At the time of proposing their metareasoning framework, Ackerman and Thompson (2017) fully acknowledged that it represented only a starting point to help catalyse further research and conceptual development in this area. However, since the inception of the framework, there has been little doubt that the concepts that it articulates have been highly influential in sparking a great deal of subsequent research and attendant theorising (e.g., Ackerman 2023; Ackerman and Thompson 2018; Mata 2020; Husselman et al. 2024; Morsanyi et al. 2019; Richardson et al. 2024; Undorf et al. 2021). In the present Special Issue, we showcase 13 articles that each contribute in important ways to the advancement of an understanding of metareasoning on the back of Ackerman and Thompson’s (2017) initial foundation and that also contribute towards the associated methodological developments in this area of enquiry.

In organising the articles in this Special Issue, we have identified three key themes around which to showcase theoretical and methodological progress. The first theme relates to the interplay between metareasoning and object-level reasoning when the latter is conceptualised from a “dual-process” perspective, whereby relatively rapid, intuitive, and heuristic processes give rise to default responses that can be intervened upon by relatively slow, reflective, and analytic processes (e.g., Evans and Stanovich 2013a, 2013b). The second theme concerns the nature of individual differences in metareasoning, which is a topic that is being subjected to increasing scrutiny. The third theme concerns the way in which metareasoning research is rapidly extending its reach to address challenging questions in new domains of enquiry beyond those that have traditionally been the preserve of investigations. As will be demonstrated, such research is giving rise both to valuable methodological developments as well as important theoretical advancements.

## 2. Metareasoning and Dual-Process Theories

The study of metareasoning has featured extensively in many recent investigations that adopt a dual-process perspective, whereby object-level reasoning is viewed as involving two qualitatively distinct types of processes, which Evans and Stanovich (2013a, 2013b) refer to as Type 1 and Type 2 processes. Type 1 processes are described as intuitive, heuristic, and associative in nature, and are defined in terms of being relatively undemanding of working-memory resources as well as autonomous (i.e., they run to completion once they have been cued). Furthermore, correlated features of Type 1 processes suggest that they are typically high capacity, rapid, non-conscious, and capable of running in parallel. In contrast, Type 2 processes are described as reflective, deliberate, analytic, and controlled, and are defined in terms of requiring working-memory resources and as having a focus on hypothetical thinking. In addition, the correlated features of Type 2 processes suggest that they tend to be slow, capacity limited, serial, and conscious. Type 2 processes are additionally considered to be less prone to biases than Type 1 processes, although they are not immune from such bases, which can arise from the application of inappropriate or inadequate analytic operations (e.g., Evans 2018; Stanovich 2018).

The interplay between Type 1 and Type 2 processes in reasoning can readily be appreciated when considering the typical responding that is observed when people tackle items that make up the cognitive reflection test (CRT; Frederick 2005; Kahneman and Frederick 2002). A good example of a CRT item is the well-known bat-and-ball problem: A bat and a ball cost $1.10 in total. The bat costs $1.00 more than the ball. How much does the ball cost? The answer of 10 cents comes to mind quickly and intuitively because of Type 1 processing but it is in fact incorrect. Deriving the correct answer, which is 5 cents, seems to depend on the reasoner resisting the default, intuitive response and instead effortfully applying reflective and analytic Type 2 processing. The Type 2 response arises for only a minority of reasoners, with the majority committing to the erroneous Type 1 answer.

Ackerman and Thompson’s (2017, 2018) metareasoning framework embraces the kind of “default-interventionist” dual-process perspective on object-level reasoning (e.g., Evans 2018; Howarth and Handley 2016) that is exemplified by people’s performance on the CRT. According to the metareasoning framework, it is only those reasoners who experience a low “feeling of rightness” in the Type 1 response who are triggered to engage in analytic Type 2 processing that can then potentially overturn the incorrect Type 1 answer. Even though Ackerman and Thompson’s metareasoning framework adopts dual-process principles, they are nonetheless careful to acknowledge that the requirement for metacognitive monitoring and control during reasoning also pertains to single-process theories, which eschew the idea that reasoning draws upon two distinct types of processes (e.g., see Kruglanski and Gigerenzer 2011). Regardless of the mechanisms that are envisaged to underpin object-level reasoning, this latter point underscores the importance of understanding the way in which people monitor rapidly generated initial answers and control the engagement of more in-depth processing. That said, the first set of articles in this Special Issue all address the interplay between metareasoning and object-level reasoning when the latter is conceptualised from a dual-process viewpoint.

In their article, Byrd et al. (2023) examine evidence for a dual-process account of reasoning by focusing on people’s performance with a purely *verbal* version of the CRT, referred to as the vCRT (originally developed and validated by Sirota et al. 2021). The vCRT is distinct from standard mathematical versions (e.g., ones that include the bat-and-ball problem) in that it is less reliant on numerical calculations. At the same time, it is conceptually equivalent to such versions in terms of being assumed to induce rapid, intuitive responses that are, in fact, incorrect, whereas correct answers presumably require reflective thinking. As an example of an item from the vCRT, consider the following: If you have only one match and you walk into a dark room where there is an oil lamp, a newspaper and wood, which thing would you light first? The intuitive “lured” answer that comes to mind quickly and effortlessly is the oil lamp. However, upon reflection, one may realise that the correct answer is the match. The vCRT has high internal consistency, high test–retest reliability, and benefits from less association with general numerical ability than the standard CRT, making it an important testbed for exploring theories of reasoning and metareasoning.

Byrd et al. (2023) set out to investigate whether performance on the vCRT aligns with a default-interventionist account. As they point out, however, this and other dual-process accounts of CRT performance have been contested in recent years based on results arising from several studies. For example, Bago and De Neys (2019) showed that most participants who answered a CRT item correctly following a period of reflection had already answered this item correctly when required to provide an initial response under extreme time pressure or under cognitive load. In addition, Stupple et al. (2017) found only a weak correlation between CRT response times and accuracy, whereas it would be expected that longer response times (indicative of more reflective processing) should be associated with increased accuracy. Item-level analyses by Stupple et al. (2017) also failed to demonstrate predicted response-time differences between correct analytic and incorrect intuitive answers for two of the three CRT items. Stupple et al.’s findings also resonate with evidence that derives from process-tracing research, which involves participants thinking aloud during the CRT. For example, Szaszi et al.’s (2017) think-aloud study indicated that most correct responses on the CRT were associated with participants articulating the correct answer from the very outset, whereas many incorrect lured responses persisted even after participants reflected on them. Such results appear to cast further doubt on a dual-process interpretation of CRT performance.

Byrd et al. (2023) report two studies using the think-aloud method, with an analysis of people’s verbalised thoughts revealing that most (but not all) correct responses involved reflection and that most (but not all) lured responses lacked reflection. These data suggest that people’s reasoning on the vCRT broadly aligns with a default-interventionist viewpoint—albeit not without some exceptions, as mentioned, which need to be accommodated in more advanced dual-process theorising. In addition, there was no evidence in Byrd et al.’s (2023) studies that thinking aloud had a disruptive effect on CRT performance when compared to a control group. This finding underscores the methodological advantages of using think-aloud protocol analysis to reveal insights regarding reasoning performance and associated metareasoning processes in cognitive reflection tests.

The next article in the Special Issue by Białek (2023) also takes a dual-process stance, but this time with a focus on the role of metacognition in debiasing reasoning and decision-making. Białek’s contribution takes the form of a review and position piece in which he explores debiasing through the lens of the “foreign language effect”, which arises when the presentation of information or instructions in a non-native language to either bilingual or multilingual participants serves to benefit their reasoning and decision-making (e.g., Keysar et al. 2012). In his article, Białek examines various proposals that have been advanced to explain the foreign language effect but finds these proposals to be inadequate given the inconsistent support that they have received. He therefore proposes an alternative working hypothesis, which is that the use of a foreign language *distorts* people’s metacognitive processing by making them less sensitive to internal cues (e.g., affective experiences), which are typically suggestive of the correctness of intuitive responses. The lack of sensitivity to such cues means that people will sometimes reflect more on problems that have intuitively appealing but incorrect solutions. This reflection facilitates the discovery of less obvious yet normatively correct solutions, resulting in improved performance. According to this model, observed improvements in judgement and decision-making are essentially a fortuitous side-effect of distorted metacognition. The author acknowledges that this working hypothesis is currently supported only by limited evidence (e.g., Białek et al. 2019; Hayakawa et al. 2017; Hennig and Hütter 2021; Muda et al. 2018). Nevertheless, if corroborated, this explanation has important implications, not only for understanding the metacognitive cues that can trigger increased analytic thinking, but also for pointing toward approaches to debias reasoning and decision-making.

In the next article, Mata (2023) adopts the CRT as a paradigm with which to examine the metacognitive awareness that people have about their reasoning performance. His particular focus is on whether people have some awareness that their thinking is flawed on those occasions where they make errors. The first two studies reported by Mata involve a methodologically innovative comparison between people’s confidence in their answers to general knowledge (GK) questions, which only require reflective thinking, versus their confidence in their answers to CRT problems, which have the potential to engender both intuitive and reflective thinking. The results showed that people are generally able to discriminate between their correct and incorrect answers, although this ability is far from perfect, and it is also greater for GK questions than for CRT problems. Interestingly, too, incorrect responses to CRT problems were found to be produced with approximately the same level of confidence, as were correct responses to GK questions. Furthermore, even though confidence was inappropriately high for incorrect responses to CRT problems, it was even higher for correct responses.

The results of these two studies are valuable in demonstrating differences in people’s confidence across tasks and across correct and incorrect responses. However, the findings also raise questions about the underlying source of these confidence differences. This issue was addressed by Mata (2023) in two further studies. In one, participants were presented with standard “conflict” versions of the CRT (where intuition and reflection are at odds), as well as “no-conflict” versions of the CRT (where there is no intuitive response and only reflective thinking can give rise to an answer). The final study was a conceptual replication using a wider range of conflict and no-conflict problems. These two studies provided clear evidence that the key factor underlying confidence differences across tasks and across correct and incorrect responses was the *conflict* that problems pose between intuition (Type 1 processing) and reflection (Type 2 processing). More specifically, overconfidence among incorrect responders was greater when problems trigger an intuitive but incorrect response. This is because, for these tricky conflict items, responders generally do not realise that an alternative, correct response is available. On the other hand, correct responders for these tricky items realise that there are alternative solutions that, whilst tempting, are incorrect.

The next article, by Ferreira et al. (2022), likewise addresses the conflict that can arise between Type 1 intuitive responses and Type 2 reflective responses of the kind that require the application of logical rules or probabilistic principles. One classic example of such a task is the so-called “lawyer–engineer problem” (Kahneman and Tversky 1972), which reads as follows:

In a study, several psychologists interviewed a group of people. The group included 5 engineers and 995 lawyers. The psychologists prepared a brief summary of their impression of each interviewee. The following description was drawn randomly from the set of descriptions: Dan is 45. He is conservative, careful, and ambitious. He shows no interest in political issues and spends most of his free time on his many hobbies, which include carpentry, sailing, and mathematical puzzles. Which of the following is more likely?

(a) Dan is an engineer;
(b) Dan is a lawyer.

When tackling this problem, people often appear to rely on the similarity between Dan’s description and the stereotype of an engineer, and thence infer that Dan is an engineer. People do this without seeming to consider the prior odds of someone being an engineer or a lawyer in this group of people (i.e., 5:995), which would make it more likely that Dan is a lawyer. What Kahneman and Tversky (1972) therefore demonstrated with this problem is that people’s judgments often seem to rely on fast and intuitive Type 1 processes (i.e., a judgement by means of so-called “representativeness”), with little evidence for this default response being overridden by slower and more effortful reflective and analytic Type 2 processes that draw upon rules of probability (i.e., a judgement that involves considering the initial base rates in the group).

In their article, Ferreira et al. (2022) suggest that the dual-process research tradition (e.g., Evans and Stanovich 2013a, 2013b; Kahneman 2003), which has been applied to explain reasoning on tasks such as the lawyer–engineer problem, may have been overly focused on Type 1 processes as the source of decision-making errors, largely ignoring the potential contribution of Type 2 processes to such errors. The authors therefore set out to uncover judgement errors specifically associated with Type 2 processing in the form of an overgeneralisation bias, whereby a reasoner continues deliberatively to apply a probabilistic rule in circumstances in which it is no longer appropriate to do so. Such an effect would be suggestive of people’s relatively superficial understanding of normative principles.

To investigate this idea experimentally, Ferreira et al. (2022) developed *rule-inadequate* versions of standard base-rate problems, in which base rates are made irrelevant. As an example, imagine that the lawyer–engineer problem included an additional premise stating that *on the first day of the study, the psychologists had randomly chosen two lawyers and two engineers to interview (from the total sample of lawyers and engineers).* In this situation, the same number of lawyers and engineers would have been interviewed, and therefore the base rates corresponding to the initial group composition (995 lawyers and 5 engineers) are not relevant, such that considering these base rates would be a mistake. Ferreira et al. (2022) conducted four experiments investigating metacognitive aspects of reasoning using both rule-adequate and rule-inadequate versions of base-rate problems. Across these studies, they observed *conflict sensitivity* (measured in terms of response latencies and response confidence) in people’s responses both to standard, rule-adequate base-rate problems and to rule-inadequate versions of these problems. People’s failure to discriminate between real and spurious conflict suggests that they often misuse statistical information and draw conclusions based on the Type 2 processing of irrelevant base rates.

Ferreira et al.’s (2022) findings have important implications for current debates regarding dual-process models of reasoning and their attendant assumptions about metareasoning. For example, with respect to the standard default-interventionist view (e.g., Evans 2018), the observation that many individuals who appear to respond normatively in standard base-rate problems are nevertheless also prone to relying on statistically irrelevant information calls into question the nature and role of Type 2 thinking in such problems. Further research is clearly needed to explore in more detail the timecourse and metareasoning components of intuitive and deliberate processing in rule-inadequate problems to clarify the theoretical implications of Ferreira et al.’s (2022) findings.

Base-rate tasks also feature in Valerjev and Dujmović’s (2023) article, which reports research that again adopts a dual-process perspective to examine the nature of conflict and metacognition in reasoning. The authors’ work builds upon a relatively recent idea in the reasoning literature, which is that people can rapidly access Type 1 “logical intuitions” when engaged in reasoning (e.g., De Neys 2012; Frey et al. 2018; Šrol and De Neys 2021). Such logical intuitions include ones that provide normatively correct solutions to mathematical, probabilistic, and logic problems. Moreover, these logical intuitions have the potential to compete with other Type 1 intuitions—more traditionally referred to as “heuristics” (e.g., matching, availability, and representativeness)—which tend to lead to non-normative and biased performance. Based on emerging evidence, the latest theorising (e.g., De Neys 2023) proposes that Type 1 processes can give rise to one or more initial intuitive responses, with the potential for a logical intuition to compete with a biased, heuristic intuition. This account provides a potential explanation of the phenomenon of base-rate neglect that is often seen in tasks such as the “lawyer–engineer problem” (Kahneman and Tversky 1972) discussed above, where people show a tendency to respond intuitively in accordance with the compelling stereotype that is presented rather than being sufficiently swayed by the conflicting base-rate information that points toward an alternative answer.

According to De Neys (2023), an interesting situation arises when two Type 1 intuitive responses are of similar strength and are in conflict, which is when a metacognitive monitoring process may trigger Type 2 activation to resolve the conflict. Indeed, many studies have shown that the presence of such conflict (together with the resulting metacognitive uncertainty that it arouses) increases the likelihood of Type 2 processing being triggered (e.g., Dujmović and Valerjev 2018; Pennycook et al. 2014, 2015; Thompson and Johnson 2014). As Valerjev and Dujmović (2023) explain, such Type 2 processing may involve the rationalisation of one of the competing responses, the consideration of additional evidence for one or both responses, or the attempt to try a new strategy and generate a novel response. In situations where metacognitive uncertainty is not at a sufficient level to trigger Type 2 processing, what is most likely is that the final response will simply be the stronger of the two Type 1 responses.

These theoretical proposals regarding the role of competing Type 1 responses in dual-process accounts of reasoning provided the inspiration for the two experiments reported by Valerjev and Dujmović (2023). These experiments involved a modification of the base-rate neglect task to enable the creation of task variants in which a belief-based Type 1 heuristic operated either in conflict or concert with another belief-based Type 1 heuristic. Moreover, Valerjev and Dujmović (2023) were able to design experiments to investigate accuracy, response time, and confidence for items where the strength of one heuristic drove responding in the opposite direction to a weaker heuristic (e.g., conflict items), as well for items where the two heuristics drove responding in the same direction (i.e., congruent items). Through sophisticated experimental manipulations, Valerjev and Dujmović demonstrate that the presence of conflict affects metareasoning, resulting in longer reasoning times and lower confidence ratings. These findings therefore favour a dual-process account of reasoning while also affirming the critical role played by metareasoning in this account.

## 3. Individual Differences in Metareasoning

The next set of papers in this Special Issue relate to the important topic of individual differences in people’s metareasoning. In general terms, research on individual differences focuses on understanding the determinants of systematic variation between people. The questions posed in this field typically pivot on the potential existence of trait-like stability within and across individuals in relation to their personalities, dispositions, and cognitive capacities—but also in relation to their metacognitive abilities. The article by Law et al. (2022) addresses head on whether individual variation in “giving-up” behaviour during cognitive task performance exhibits such trait-like properties. According to Ackerman and Thompson’s (2017, 2018) metareasoning framework, the process of giving up when a solution may not seem achievable reflects an adaptive metacognitive control decision, whereby individuals opt-out of responding to mitigate resource costs and to avoid making erroneous responses (Payne and Duggan 2011). However, as Law et al. (2022) note, research is needed to determine whether individuals systematically vary in this giving-up behaviour across different tasks. If this is indeed the case, then it is also vital to ascertain which variables are meaningfully associated with this behaviour.

In addressing these questions, Law et al. (2022) deployed three different uncertainty-monitoring paradigms that depend on perceptual decision-making, including one from research on animal metacognition, referred to as the sparse–uncertain–dense (SUD) task. In this task, participants are provided with boxes of varying degrees of pixel density and are required to decide whether a box is sparse, dense or whether they are uncertain, with the latter option reflecting an opt-out decision. Using these kinds of task-based methodological innovations, the authors report a study that not only established the factorial stability in people’s giving-up tendencies but also supported the assumed adaptive nature of such tendencies by showing how the giving-up factor correlates positively with cognitive ability, rational decision-making, and academic performance. This research provides a valuable basis for further metareasoning studies to investigate the characteristics and underlying processes of this trait-like giving-up construct as well as the critical role that it plays in learning, cognitive processing, and decision-making.

The theme of individual differences in metareasoning is also central to the article by Morsanyi and Hamilton (2023), who report a study of the development of intuitive and analytic thinking in autism. When reasoning, it is often of critical importance for a person to be able to engage in metacognitive control to resist adhering to an initial, default intuitive response, and instead to apply more effortful and reflective analytic thinking that can potentially lead to the generation of a normatively correct response (e.g., Evans and Stanovich 2013a, 2013b). In their study, Morsanyi and Hamilton aimed to investigate whether closely matched autistic and neurotypical individuals share the same normatively incorrect intuitions that typically dominate responses to the cognitive reflection test (CRT; Frederick 2005; Kahneman and Frederick 2002), or whether individuals with autism show increased analytic processing, as suggested by some researchers (e.g., De Martino et al. 2008). The CRT represents a good testbed for investigating the balance of intuitive versus analytic processes that arise in autistic and neurotypical individuals, given that the problems that make up the test tend to elicit an intuitive but incorrect response, whereas the correct response requires effortful reflection.

Morsanyi and Hamilton’s (2023) study included adolescents and young adults and involved methodological improvements on previous studies investigating CRT performance in autism. One such improvement was the use of the 6-item CRT-Long (Primi et al. 2016), which measures cognitive reflection more effectively in younger individuals, who often score zero on the original CRT. Like previous findings with the CRT, Morsanyi and Hamilton’s (2023) results showed an age-related increase in analytic responding and a decrease in intuitive responding. Importantly, however, the proportion of both intuitive and analytic responses across autistic and neurotypical participants was identical in both age groups, suggesting similar object-level processing as well as similar levels of metacognitive control. As the authors acknowledge, finding no group differences in cognitive reflection does not necessarily imply that autistic and neurotypical samples would show an absence of differences on other reasoning or decision-making tasks. As such, more research is needed on this issue, including investigations that delve deeper into the metacognitive monitoring and control processes that accompany both intuitive and analytic responding in autism.

In the next article in this Special Issue, Stanovich and Toplak (2023) also deal with the topic of individual differences, this time in relation to the thinking disposition that is referred to as “actively open-minded thinking” (AOT). This construct is measured by means of items that tap reflective thought, the willingness to consider alternative opinions, sensitivity to evidence that is contradictory to current beliefs, and the willingness to postpone closure. AOT scales strongly predict performance on heuristics and biases tasks (e.g., Stanovich and West 2000) as well as the avoidance of reasoning traps, such as belief in conspiracy theories (e.g., Stanovich et al. 2016). As Stanovich and Toplak (2023) note, AOT has much conceptual overlap with metareasoning processes, both through its connection with notions of goal management and epistemic self-regulation (e.g., by means of monitoring ongoing thinking, allocating cognitive resources, and attentional switching) and via the concept of cognitive decoupling (i.e., the inhibitory override of intuitive responses together with the engagement of hypothetical reasoning and cognitive simulation, which are necessary to compute normatively correct responses).

In their article, Stanovich and Toplak (2023) not only provide a valuable review of their 25-year history of studying AOT and developing AOT measurement scales, but they also present their new, brief 13-item AOT scale, which addresses prior criticisms while building upon previous refinements. In discussing why AOT scales are such good predictors of performance on heuristics and biases tasks, the authors conclude that it is because these scales tap important processes of cognitive decoupling and decontextualisation that modernity increasingly requires (see Stanovich 2004)—processes that appear to be inherently tied to effective metareasoning, such as checking the validity of intuitive responses (cf. De Neys and Pennycook 2019; Pennycook et al. 2015). However, one paradox remains, and is discussed at length by Stanovich and Toplak, which is why it is that AOT scales are potent predictors of performance on most rational thinking tasks, yet they do not predict the attenuation of “myside bias”—the tendency to generate and evaluate evidence in a manner favourable toward one’s prior opinions and attitudes. This consistent observation is disconcerting, because of all the biases that one would expect to be negatively correlated with AOT, myside bias is the primary one. Stanovich and Toplak present several pointers toward future research that might unravel the mystery of this absent correlation between AOT and the avoidance of myside bias, such as failures of metacognitive monitoring (e.g., the recognition of alternative beliefs). This topic seems ripe for further research, which could not be more timely or important for effective human reasoning in the modern world.

## 4. Extending the Reach of Metareasoning Research

Metareasoning research has been expanding rapidly in recent years and is continually extending its reach to address challenging issues in domains that range beyond the traditional ones that informed the development of Ackerman and Thompson’s (2017, 2018) original metareasoning framework. Such traditional domains of enquiry feature in all the aforementioned articles, with their focus on deductive and probabilistic tasks as well as the CRT. In this section of the Special Issue, however, the scope broadens to consider metareasoning research that is taking place in domains beyond this established remit. As will be shown, such research is engendering valuable methodological developments, in addition to spearheading important conceptual advancements.

As a case in point, Shen et al. (2022) undertook a spectral electroencephalography (EEG) analysis to investigate the neural correlates of people being in a tip-of-the-tongue (TOT) state, which is a spontaneously occurring metacognitive state that is indicative of an answer to a query being almost at hand but not quite forthcoming. This TOT state is akin to what Ackerman and Thompson (2017, 2018) refer to as an initial “judgment of solvability”, when people are presented with a question or problem. This judgement can trigger metacognitive control processes that deploy resources as the individual strives toward achieving an answer to the question at hand or a solution to a given problem. Indeed, although such TOT states are imbued with a sense of frustration, they are also associated with intense curiosity and a strong desire to find a resolution to the query, which translates into increased goal-directed cognitive processing (e.g., Metcalfe and Jacobs 2024; Schwartz and Metcalfe 2011).

In their study, Shen et al. (2022) showed that if an individual was in a TOT state when presented with a verbal query, then alpha suppression was in evidence in the spectral EEG analysis across centro-parietal regions prior to the experimenter’s presentation of resolving feedback (note that the alpha frequency band ranges between 8 Hz and 12 Hz). Additional analyses also indicated that the occurrence of alpha suppression was associated with participants’ verbal affirmations of being in a TOT state. Interestingly, too, Shen et al. (2022) observed that the opposite of alpha suppression in the centro-parietal regions—that is, alpha expression in these same regions—was associated with the feeling of “not knowing” the answer to a query. An increase in alpha power has often been interpreted as indicating reduced information processing and of being associated with so-called “cortical idling” and corresponding mental idling (Pfurtscheller 1999) as well as mind wandering (e.g., Baldwin et al. 2017). Overall, Shen et al.’s (2022) findings are important, as they are highly suggestive of alpha suppression being a neural signature of a spontaneous, conscious, and goal-directed metacognitive state of awareness. In this respect, it is noteworthy that the suppression of alpha band activity has traditionally been related to enhanced attention and more engaged information processing (Neuper and Pfurtscheller 2001; Pfurtscheller and Lopes da Silva 1999). The identification of neural markers of metacognition clearly represents an exciting and fruitful line of research for the future.

In the aforementioned article, Shen et al. (2022) note close similarities between the situation where a person is in a TOT state when trying to answer a given query and the situation where a problem-solver is currently unable to solve a problem but nevertheless has a high degree of certainty that a solution is within reach (cf. Polanyi 1974). In the next article in the Special Issue, Graf et al. (2023) focus on problems of a different type, whereby an initial, incorrect mental representation is triggered that needs to be restructured to enable the problem solver to find the solution. When attempting these so-called “insight problems”, people often reach a point of “impasse”, such that they have no sense that they can progress toward a solution. A period of impasse will sometimes then be followed by a sudden restructuring process or “representational change”, which invokes an immediate insight into how the problem can successfully be solved (e.g., see Bowden et al. 2005; Ohlsson 2011). Restructuring reflects a change in strategy, which is presumably initiated by a metacognitive control process that is itself triggered by the metacognitive monitoring system identifying that no progress is being made with a current strategy. As Graf et al. (2023) point out, however, despite the theoretical assumption that this restructuring process happens suddenly with insight problems (engendering a typical “Aha!” experience), the evidence for this assumption remains inconsistent and inconclusive. Arguably, one key reason for this lack of clarity relates to the fact that most measures of restructuring and insight rely on the problem-solver’s subjective experience of aspects of their own solution process. As such, a more *objective* measure of metacognition and the occurrence of sudden restructuring would be welcome.

Graf et al. (2023) propose that one way to obtain such objective data relating to problem restructuring and the occurrence of insight is to trace problem-solving processes using eye-movement tracking. Previous research using eye tracking to investigate restructuring processes in insight problem solving has employed matchstick arithmetic problems, which use matchsticks to present false arithmetic statements (written using Roman numerals, arithmetic operators, and equal signs), with the participant’s task being to transform the false arithmetic statement into a true statement by moving only one stick (Knoblich et al. 2001). Four types of matchstick arithmetic problems have been defined, which involve varying levels of difficulty, depending on the constraints that need to be relaxed and the tightness of the chunks that need to be decomposed. Knoblich et al.’s eye-tracking study (Knoblich et al. 2001) provided good evidence that people initially fixate on elements of problems that are irrelevant to a solution (suggestive of an initial incorrect problem representation based on an effective solution strategy), with solvers only shifting their visual attention to relevant elements of problems in the final third of the problem-solving period (suggestive of a change in strategy and the construction of a more effective representation).

As Graf et al. (2023) note in their article, although Knoblich et al.’s (2001) study provides compelling evidence for the view that a restructuring of the problem representation takes place in problems that require constraint relaxation, what this previous research fails to show is whether this change is sudden or gradual. In their reported research, Graf et al. (2023) re-analyse their own previous eye-tracking data (from Bilalić et al. 2019) and demonstrate convincingly that it is possible to identify the occurrence of abrupt representational changes in the form of sudden bursts of attention to key aspects of a problem—albeit only through the application of nonlinear statistical models in conjunction with fine-grained change-point analysis. Additionally, they show that the provision of explicit solution hints to participants who had not solved a problem after five minutes served to reorient the focus of attention, demonstrably changing the dynamics of restructuring in insight problem solving. In sum, although an insight problem may well require the sudden restructuring of an initial mental representation, driven by metacognitive cues, it is only sophisticated analytical and statistical approaches that can uncover such strategic restructuring events.

In the next article, Kenett et al. (2023) maintain Graf et al.’s focus on the under-researched role of metacognitive cues in creative cognition, albeit not in relation to insight problem solving, but rather with respect to creative idea generation using the alternative uses task (AUT, e.g., Guilford 1967), whereby people are asked to come up with as many alternative uses as they can for common objects such as a bucket. Kenett et al. were specifically concerned with the heuristic cues that underlie people’s metacognitive judgments of the *originality* of their ideas, with their particular research interest being on the role played by the “semantic distance” between concepts in informing people’s originality judgments. Semantic distance is a measure derived from the computational analysis of large textual corpora, and it quantifies the conceptual *dis-similarity* between the AUT object and the words that are present in the open-ended responses that participants generate when coming up with alternative uses for that object (e.g., see Beaty and Johnson 2021).

In their research, Kenett et al. (2023) examined the extent to which semantic distance contributes explanatory value in predicting objective originality scores (i.e., the frequency with which an idea is generated within a sample of participants) as well as in predicting subjective originality judgments (i.e., metacognitive self-assessments of originality). In two experiments (one of which re-analysed previous data from Sidi et al. 2020), Kenett et al. found that semantic distance was a strong predictor of both objective originality scores and subjective originality judgments, while also being a cue that consistently predicted originality scores more strongly than originality judgments (i.e., people tend to *underestimate* their originality). Systematic priming manipulations that Kenett et al. built into their second experiment also revealed that the extent to which semantic distance can influence originality judgments is highly malleable and context dependent. Overall, this study highlights the role of semantic distance as a previously unacknowledged metacognitive cue that has the power to influence originality judgments in creative idea generation.

The next article in the Special Issue by Caro et al. (2022) focuses on metareasoning in machines, such as artificially intelligent systems, rather than in humans, representing a seemingly marked departure from the preceding articles. What is abundantly clear in Caro et al.’s reported work, however, is that many of the conceptual challenges that arise when considering metareasoning in non-human intelligent systems have close parallels in the domain of human intelligence. As these authors note, metareasoning research suffers from the “heterogeneity problem”, whereby different researchers across different domains (e.g., psychology, education, computer science, artificial intelligence, and engineering) build diverse metareasoning models for intelligent systems that have comparable functionality but that use ambiguous terminology and contradictory descriptions as well as non-standardised conventions.

To address the heterogeneity problem, Caro et al. (2022) present an ontology-driven knowledge representation for metareasoning in the domain of artificial intelligence that is extendible to all intelligent systems, which they refer to as IM-Onto. This proposed ontology provides a visual means of sharing a common understanding of the structure and relationships between metareasoning terms and concepts. In their research, the authors also adopted a rigorous research method to ensure that the two main requirements of as ontology were met: integrity based on relevant knowledge and acceptance by researchers and practitioners. The data-driven evaluation of the ontology revealed a high accuracy rate, indicating that many of the knowledge elements in the ontology provide useful information for understanding metareasoning in intelligent systems. This research is important in providing a valuable stepping-stone toward a consistent ontology that can be used for sharing a common understanding of the structure and relationships among terms and concepts related to metareasoning across all intelligent systems, whether artificial or human.

The final article in the Special Issue by Richardson and Ball (2024) addresses a significant challenge for current metareasoning research, which relates to the fact that it is almost exclusively focused on explaining the monitoring and control processes that arise at the level of *individual* reasoners, with little consideration being given to the metareasoning that occurs when two or more individuals work conjointly on a reasoning task. Richardson and Ball argue that this omission in the metareasoning literature is significant, given the many domains in which team-based reasoning is critical, such as those that relate to design, innovation, process control, and defence and security. In their article, Richardson and Ball (2024) tackle the absence of a conceptual framework head on to address the nature of team metareasoning by discussing how Ackerman and Thompson’s (2017) metareasoning framework can be extended to situations where people reason collaboratively to reach an agreed solution to a problem or to decide on a common course of action.

In developing a collaborative metareasoning framework, Richardson and Ball (2024) expand upon an important tripartite distinction relating to monitoring processes, which they have previously advanced in the literature (Richardson et al. 2024; see also Pickering and Garrod 2021, who independently drew an equivalent distinction). This distinction aims to capture the difference between “self monitoring” (an individual’s monitoring of their own performance), “other monitoring” (an individual’s monitoring of the performance of others) and “joint monitoring” (the unified monitoring of collective performance). The tripartite distinction gives rise to many important questions regarding the kinds of cues that team members draw upon when engaging in other monitoring and joint monitoring, which are questions that Richardson and Ball (2024) address extensively in their article. Importantly, too, they propose a parallel tripartite distinction for control processes that can arise in teams. In articulating this novel distinction, the authors differentiate between “self-focused control” (i.e., an individual’s procedural decisions about how to progress or terminate their own reasoning), “other-focused control” (i.e., an individual’s procedural decisions about how to control the performance of others) and “joint control” (i.e., the unified control of procedural decisions regarding how to advance or terminate collective performance).

Richardson and Ball (2024) discuss in detail the nature of the control processes that operate at these three aforementioned levels. Their notion of other-focused control seems particularly fascinating in the context of collaborative metareasoning, as it captures the way in which people engage in metareasoning to persuade others through argumentation and negotiation. These various forms of other-focused control—which may even involve deception—can all be highly profitable for team progress toward a good outcome, such as when progress is stalling because of conflicted viewpoints. Indeed, the capacity to exert effective other-focused control over a team’s ongoing reasoning has been shown to be an important aspect of a team leader’s metareasoning repertoire (e.g., Ball and Ormerod 2000). In elaborating upon the distinct types of metacognitive monitoring and control that seem to pervade team-based reasoning, Richardson and Ball (2024) also discuss the prospect for developing a comprehensive collaborative metareasoning framework that centres on the importance of changes in team members’ *language* as a means to track metacognitive uncertainty and misalignment over time. Such language-focused metacognitive tracking has significant potential to inform both theoretical developments and applied interventions to enhance team-based reasoning and decision-making performance.

## 5. Conclusions

The 13 articles in this Special Issue provide an exciting snapshot of current theoretical and methodological developments in the rapidly expanding field of metareasoning research. Many of the articles address the close links that exist between metareasoning concepts and dual-process accounts of reasoning, with new developments in dual-process theorising giving fresh impetus to a consideration of the role of metacognitive monitoring and control processes in many established reasoning, decision-making and problem-solving paradigms. We have also showcased articles that address the increasingly important topic of individual differences in people’s metareasoning skills. There is clearly much value to be had in gaining a deeper understanding of the ways in which metareasoning varies in its functioning and effectiveness across individuals. In particular, such an understanding can inform the design and development of tailored approaches and interventions to support the training of metareasoning skills in ways that can enable the generation of sound inferences, improved decisions, and enhanced solutions. Finally, we have included a set of articles in the Special Issue that demonstrate how metareasoning research is addressing challenging questions in new domains beyond those that have traditionally been the focus of enquiry, such as in studying metareasoning in the tip-of-the-tongue phenomenon, in creative cognition, in artificial systems, and in team collaboration. Overall, we are in no doubt that metareasoning research is extremely vibrant and is constantly engendering valuable methodological developments and theoretical advancements.
